# Numerical Study of Natural Convection Heat Transfer in a Porous Annulus Filled with a Cu-Nanofluid

**DOI:** 10.3390/nano11040990

**Published:** 2021-04-12

**Authors:** Lingyun Zhang, Yupeng Hu, Minghai Li

**Affiliations:** 1 School of Aerospace Engineering, Beijing Institute of Technology, Beijing 100000, China; zhanglingyun17@gscaep.ac.cn; 2 Institute of System Engineering, China Academy of Engineering Physics, Mianyang 621999, China

**Keywords:** natural convection, heat transfer, water-based nanofluid, Brownian motion, porous medium, Darcy–Brinkman equation, numerical simulation

## Abstract

Natural convection heat transfer in a porous annulus filled with a Cu nanofluid has been investigated numerically. The Darcy–Brinkman and the energy transport equations are employed to describe the nanofluid motion and the heat transfer in the porous medium. Numerical results including the isotherms, streamlines, and heat transfer rate are obtained under the following parameters: Brownian motion, Rayleigh number (10^3^–10^5^), Darcy number (10^−4^–10^−2^), nanoparticle volume fraction (0.01–0.09), nanoparticle diameter (10–90 nm), porosity (0.1–0.9), and radius ratio (1.1–10). Results show that Brownian motion should be considered. The nanoparticle volume fraction has a positive effect on the heat transfer rate, especially with high Rayleigh number and Darcy number, while the nanoparticle diameter has an inverse influence. The heat transfer rate is enhanced with the increase of porosity. The radius ratio has a significant influence on the isotherms, streamlines, and heat transfer rate, and the rate is greatly enhanced with the increase of radius ratio.

## 1. Introduction

Fluid flow and heat transfer due to natural convection in porous annulus is one of the most considerable research issues due to its wide applications in science and engineering [1,2,3], such as thermal insulators, chemical catalytic convectors, thermal storage systems, geothermal energy utilization, electronic cooling, and nuclear reactor systems. Therefore, natural convection in porous annulus has been extensively investigated during the past decades. Caltagirone [4] was the first to study natural convection in a saturated porous medium bounded by two concentric, horizontal, isothermal cylinders experimentally and numerically. The author used the Christiansen effect in order to visualize a fluctuating three-dimensional thermal field for Rayleigh number exceeding some critical value. Rao et al. [5,6] performed steady and transient investigations on natural convection in a horizontal porous annulus heated from the inner face using Galerkin method. The effects of Rayleigh number and Darcy number on heat transfer characteristics were studied. In addition, the bifurcation point was obtained numerically, which compared very well with that from experimental observation.

Himasekhar [7] examined the two-dimensional bifurcation phenomena in thermal convection in horizontal, concentric annuli containing saturated porous media. The fluid motion is described by the Darcy–Oberbeck–Boussinesq equations, which were solved using regular perturbation expansion. The flow structure was obtained under different parameters, such as the radius ratio and Rayleigh–Darcy number. A parametric study was performed by Leong et al. [8] to investigate the effects of Rayleigh number, Darcy number, porous sleeve thickness, and relative thermal conductivity on heat transfer characteristics. Braga et al. [9] presented numerical computations for laminar and turbulent natural convection within a horizontal cylindrical annulus filled with a fluid saturated porous medium. Computations covered the range 25 < *Ra*_m_ < 500 and 3.2 × 10^−4^ > *Da* > 3.2 × 10^−6^ and made use of the finite volume method. Khanafer et al. [10] carried out a numerical simulation in order to examine the parametric effects of Rayleigh number and radius ratio on the role played by natural convection heat transfer in the porous annuli. The model was governed by Darcy–Oberbeck–Boussinesq equations and solved using the Galerkin method. In order to investigate the buoyancy-induced flow as affected by the presence of the porous layer, Alloui and Vasseur [11] studied natural convection in a horizontal annular porous layer filled with a binary fluid under the influence of the Soret effect using the Darcy model with the Boussinesq approximation. Numerical solutions of the full governing equations are obtained for a wide range of the governing parameters, such as Rayleigh number, Lewis number, buoyancy ratio, radius ratio of the cavity, and normalized porosity.

Belabid and Cheddadi [12] solved natural convection heat transfer within a two-dimensional horizontal annulus filled with a saturated porous medium using ADI (Alternating Direction Implicit) finite difference method. This work placed emphasis on the mesh effect on the determination of the bifurcation point between monocellular and bicellular flows for different values of the aspect ratio. In a recent work, Belabid and Allali [13,14] studied the effects of a periodic gravitational and temperature modulation on the convective instability in a horizontal porous annulus. Results showed that the convective instability is influenced by the amplitude and the frequency of the modulation. Rostami et al. [15] provided a review of recent natural convection studies, including experimental and numerical studies. The effects of the parameters, such as nanoparticle addition, magnetic fields, and porous medium on the natural convection were examined.

The traditional fluids in engineering, such as water and mineral oils, have a primary limitation in the enhancement of heat transfer due to a rather low thermal conductivity. The term nanofluids, which was first put forward by Choi [16], is used to describe the mixture of nanoparticles and base fluid. Due to the relatively higher thermal conductivities, nanofluids are considered as an effective approach to meet some challenges associated with the traditional fluids [17]. In the last few decades, studies on the natural convection of nanofluids in enclosure were conducted by a number of researchers [18]. For instance, Jou and Tzeng [19] investigated the numerically natural convection heat transfer enhancement utilizing nanofluids in a two-dimensional enclosure. Results showed that increasing the buoyancy parameter and volume fraction of nanofluids caused an increase in the average heat transfer coefficient. Ghasemi et al. [20] simulated the natural convection heat transfer in an inclined enclosure filled with a CuO–water nanofluid. The effects of pertinent parameters such as Rayleigh number, inclination angle, and solid volume fraction on the heat transfer characteristics were studied. The results indicated that the heat transfer rate is maximized at a specific inclination angle depending on Rayleigh number and solid volume fraction.

In a related work, Abu-Nada and Oztop [21] found that the effect of nanoparticles concentration on Nusselt number was more pronounced at low volume fraction than at high volume fraction and the inclination angle could be a control parameter for nanofluid filled enclosure. Soleimani et al. [22] studied the natural convection heat transfer in a semi-annulus enclosure filled with a Cu–water nanofluid using the Control Volume based Finite Element Method. The numerical investigation was carried out for different governing parameters, such as Rayleigh number, nanoparticle volume fraction, and angle of turn for the enclosure. The results revealed that there was an optimal angle of turn in which the average Nusselt number was maximum for each Rayleigh number. Seyyedi et al. [23] simulated the natural convection heat transfer of Cu–water nanofluid in an annulus enclosure using the Control Volume-based Finite Element Method. The Maxwell–Garnetts and Brinkman models were employed to estimate the effect of thermal conductivity and viscosity of nanofluid. The results showed the effects of the governing parameters on the local Nusselt number, average Nusselt number, streamlines, and isotherms. Boualit et al. [24] and Liao [25] studied respectively natural convection heat transfer of Cu- and Al_2_O_3_-water nanofluids in a square enclosure under the horizontal temperature gradient. Both the flow structure and the corresponding heat transfer characteristics at different Rayleigh numbers and nanoparticle volume fractions were obtained. Wang et al. [26] investigated numerically the natural convection in a partially heated enclosure filled with Al_2_O_3_ nanofluids. The results indicated that at low Rayleigh numbers, the heat transfer performance increased with nanoparticle volume fraction, while at high Rayleigh numbers, there existed an optimal volume fraction at which the heat transfer performance had a peak. In a recent work, Mi et al. [27] examined the effects of graphene nano-sheets (GNs) nanoparticles by comparing the thermal conductivity of graphene nano-sheets (GNs)/ethylene glycol (EG) nanofluid with EG thermal conductivity. Results showed that the presence of nanoparticles improved the thermal conductivity, and with increasing temperature, the effect of adding GNs was strengthened.

Although several numerical and experimental studies on the natural convection heat transfer were published, most of them concentrated on traditional fluids in cavities, and only a few of them consider a nanofluid in a porous annulus [28,29,30]. In the present work, steady natural convection heat transfer in a porous annulus filled with a Cu-nanofluid has been investigated, and the governing equations, including the Darcy–Brinkman equation, were solved using the Galerkin method. This paper presented a systematical examination on the effects of Brownian motion, solid volume fraction, nanoparticle diameter, Rayleigh number, Darcy number, porosity on the flow pattern, temperature distribution, and heat transfer characteristics. To the best of our knowledge, no study on this problem has been considered before, and accordingly, the current paper will address this topic.

## 2. Problem Formulation

### 2.1. Physical Description

**Figure 1:**
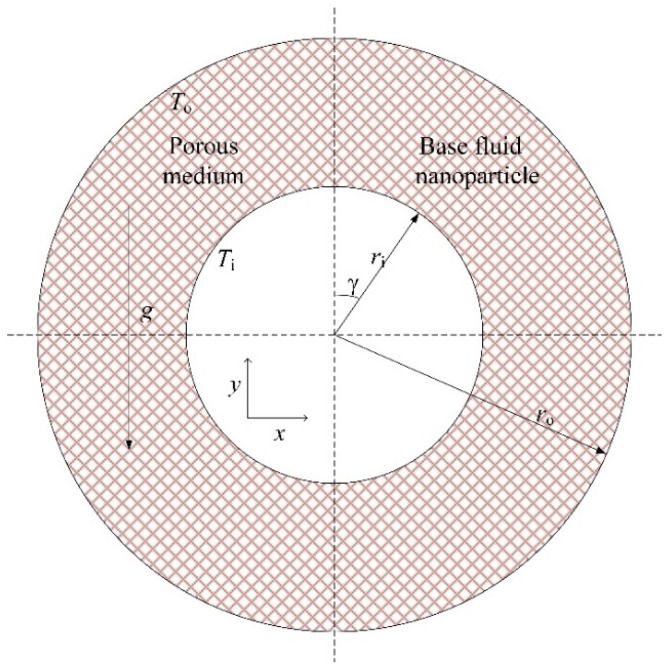
Sketch of problem geometry.

We consider porous annulus filled with a Cu–water nanofluid between a horizontal inner and outer cylinder of radius *r*_i_ and *r*_o_, respectively, as shown in Figure 1. The inner and outer cylinders are kept at uniform high temperature *T*_i_ and low temperature *T*_o_, respectively. It is taken into consideration that the flow is two-dimensional, steady, and laminar due to the low velocity. The porous medium is considered as isotropic, homogeneous, and filled with a nanofluid, which is thermal equilibrium with the solid matrix, and the Darcy–Brinkman equation without inertia item is adopted. For the nanofluid, the effect of Brownian motion [31,32,33] is considered. The thermophysical properties of the base water, copper nanoparticles, and solid structure of the porous medium used in this study are presented in Table 1 and Table 2 [34,35].

### 2.2. Governing Equations and Boundary Conditions

By introducing the Boussinesq approximation, the governing equations for the heat transfer and fluid flow can be written as follows:

(1) $$\frac{\partial u}{\partial x}+\frac{\partial v}{\partial y}=0$$

(2) $$\left(u\frac{\partial u}{\partial x}+v\frac{\partial u}{\partial y}\right)=-\varepsilon \frac{1}{\rho_{\mathrm{nf}}}\frac{\partial p}{\partial x}+\varepsilon \nu_{\mathrm{nf}}\left(\frac{\partial^{2}u}{\partial x^{2}}+\frac{\partial^{2}u}{\partial y^{2}}\right)-\varepsilon^{2}\frac{\nu_{\mathrm{nf}}}{K}u$$

(3) $$\left(u\frac{\partial v}{\partial x}+v\frac{\partial v}{\partial y}\right)=-\varepsilon \frac{1}{\rho_{\mathrm{nf}}}\frac{\partial p}{\partial y}+\varepsilon \nu_{\mathrm{nf}}\left(\frac{\partial^{2}v}{\partial x^{2}}+\frac{\partial^{2}v}{\partial y^{2}}\right)-\varepsilon^{2}\frac{\nu_{\mathrm{nf}}}{K}v+\varepsilon^{2}g\beta \left(T-T_{o}\right)$$

(4) $${\left(\rho c_{p}\right)}_{\mathrm{nf}}\left(u\frac{\partial T}{\partial x}+v\frac{\partial T}{\partial y}\right)=k_{\mathrm{mnf}}\left(\frac{\partial^{2}T}{\partial x^{2}}+\frac{\partial^{2}T}{\partial y^{2}}\right)$$

where (*x*, *y*) are the Cartesian coordinates of the geometry, (*u*, *v*) are the velocity components, *T* and *p* are the temperature and pressure, respectively, *ρ*, (*ρc*_p_), *ν*, *β* and *α* denote the density, heat capacitance, kinematic viscosity, thermal expansion coefficient, and thermal diffusion, respectively, *k* is the thermal conductivity, *ε* is the porosity, and *K* is the medium permeability. The subscripts nf and mnf designate nanofluid and porous medium filled with nanofluid.

The effective thermal conductivity of the porous medium filled with nanofluid can be as modeled as:

(5) $$k_{\mathrm{mnf}}=\varepsilon k_{\mathrm{nf}}+\left(1-\varepsilon \right)k_{s}.$$

Using the following dimensional variables,

$\left(X,Y\right)=\frac{\left(x,y\right)}{\left(r_{o}-r_{i}\right)}$, $\left(U,V\right)=\frac{\left(u,v\right)\left(r_{o}-r_{i}\right)}{\alpha_{\mathrm{mnf}}}$, $P=\frac{p{\left(r_{o}-r_{i}\right)}^{2}}{\rho_{\mathrm{nf}}\alpha_{\mathrm{mnf}}{}^{2}}$, $\theta =\frac{T-T_{o}}{T_{i}-T_{o}}$, $\alpha_{\mathrm{mnf}}=\frac{k_{\mathrm{mnf}}}{{\left(\rho c_{p}\right)}_{\mathrm{nf}}}$, $Ra=\frac{g\beta \left(T_{i}-T_{o}\right){\left(r_{o}-r_{i}\right)}^{3}}{v_{\mathrm{nf}}\alpha_{\mathrm{mnf}}}$, ${Pr}_{}=\frac{v_{\mathrm{nf}}}{\alpha_{\mathrm{mnf}}}$, $Da=\frac{k}{L^{2}}$.

The governing Equations (1)–(4) reduce to a dimensionless form:

(6) $$\frac{\partial U}{\partial X}+\frac{\partial U}{\partial Y}=0$$

(7) $$U\frac{\partial U}{\partial X}+V\frac{\partial U}{\partial Y}=-\varepsilon \frac{\partial P}{\partial X}+\varepsilon Pr\left(\frac{\partial^{2}U}{\partial X^{2}}+\frac{\partial^{2}U}{\partial Y^{2}}\right)-\varepsilon^{2}\frac{Pr}{Da}U$$

(8) $$U\frac{\partial V}{\partial X}+V\frac{\partial V}{\partial Y}=-\varepsilon \frac{\partial P}{\partial X}+\varepsilon Pr\left(\frac{\partial^{2}V}{\partial X^{2}}+\frac{\partial^{2}V}{\partial Y^{2}}\right)-\varepsilon^{2}\frac{Pr}{Da}U+\varepsilon^{2}RaPr\theta$$

(9) $$U\frac{\partial \theta }{\partial X}+V\frac{\partial \theta }{\partial Y}=\frac{1}{Pr}\left(\frac{\partial^{2}\theta }{\partial X^{2}}+\frac{\partial^{2}\theta }{\partial Y^{2}}\right)$$

where (*X*, *Y*) are the dimensionless Cartesian coordinates of the geometry, (*U*, *V*) are the dimensionless velocity components, and *θ* and *P* are the temperature and pressure, respectively. *Ra*, *Pr*, and *Da* denote respectively the Raleigh number, Prandtl number, and Darcy number.

The boundary conditions for this problem are as follows:

On the outer cylinder surface:

(10) θ=0.

On the inner cylinder surface:

(11) θ=1.

On the outer and inner cylinder surfaces:

(12) U=V=0.

The local and overall heat transfer rate along the inner cylinder surface are estimated using the local Nusselt number and average Nusselt number, respectively:

(13) $$Nu_{\mathrm{loc}}={\frac{\partial \theta }{\partial N}|}_{S}=\sqrt{{\left(\frac{\partial \theta }{\partial X}\right)}^{2}+{\left(\frac{\partial \theta }{\partial Y}\right)}^{2}}$$

(14) $$Nu_{\mathrm{avg}}=\frac{1}{S}\int_{0}^{s}Nu_{\mathrm{loc}}$$

where *S* is the non-dimensional length along the inner cylinder surface.

## 3. Numerical Procedure

The dimensionless governing Equations (6)–(9) together with the boundary conditions (10) and (11) have been solved numerically using the commercial software tool, which is known as COMSOL Multiphysics (Version 5.5, COMSOL Inc., Stockholm, Sweden). The software employs the Galerkin finite element method, which enforces the orthogonality of residuals to all basis functions in a basis. In Galerkin formulation, weighting functions are chosen to become identical to basis functions [36]. In this paper, we have employed a segregated and parallel direct (Pardiso) solver to solve those equations. As convergence criteria, 10^−6^ has been chosen for all dependent variables.

### 3.1. Grid Generation and Independence Test

**Figure 2:**
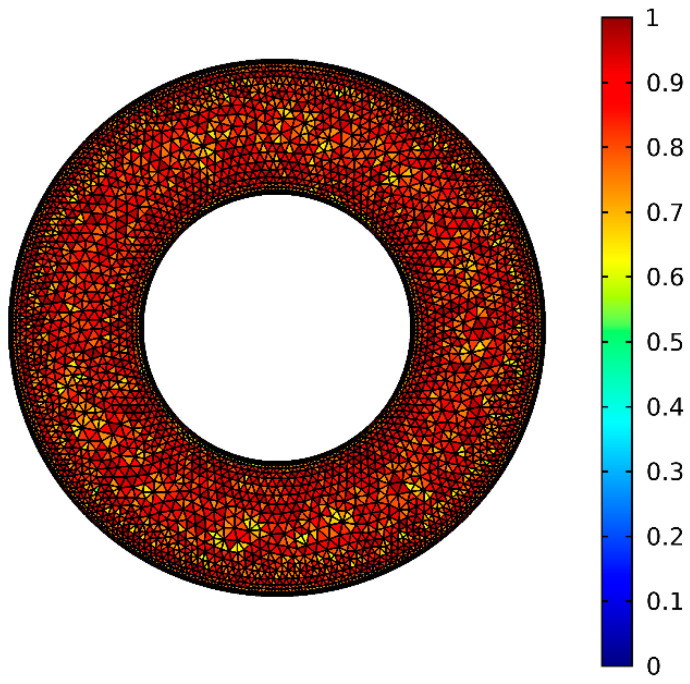
Grid generation of the structure with a legend of quality measure.

In the finite element method, grid generation is the technique to discretize the computational domain into subdomains. In this study, we have adopted unstructured triangular elements in the interior and structured quadrilateral elements on the boundary. Figure 2 is the grid generation of the structure with a legend of quality measure. A quality of 1 represents the best possible grid quality, and a quality of 0 represents the worst possible grid quality. For purpose of ensuring the grid independence of the numerical solution, different grid levels in the Comsol Multiphysics are examined. As shown in Table 3, the average Nusselt number of on the inner cylinder at different grids is presented for Cu nanofluid when the nanoparticle volume fraction (*ϕ*) is 0.5, nanoparticle diameter (*d*_sp_) is 50 nm, Rayleigh number (*Ra*) is 10^5^, porosity (*ε*) is 0.5, and Darcy number (*Da*) is 10^−2^. The difference between normal and extremely fine is within 1.35%. By comprehensive considering the calculation accuracy and cost, the extra fine level is chosen in this study.

### 3.2. Code Validation

Due to the lack of experimental data for conjugate heat transfer of nanofluid in an annulus filled with porous medium, we have compared our results with the numerical results of Abhishek Kumar Singh and Tanmay Basak et al. [37] for a square cavity filled with base fluid (*ϕ =* 0). The comparisons are presented in Table 4, when *ε* = 0.4, and the deviations are 0%, 0%, and 0.84% with *Ra* = 10^3^, 10^4^, and 10^5^, respectively. When *ε* = 0.9, the deviations are 0.5%, 1.2%, and 1.3% with *Ra* = 10^3^, 10^4^, and 10^5^, respectively. It is clear that the current results are in good agreement with the earlier work, and the maximum deviation is 1.3%. In addition, it can be seen that the deviation is increased with the increase of Rayleigh number and porosity. The validation work has enhanced the confidence in the numerical solution of the current study.

## 4. Results and Discussion

In this section, numerical simulations are carried out to investigate the flow and heat transfer characteristics of the nanofluid filled in the porous annulus. The results present the effects of several parameters, such as Brownian motion, nanoparticle diameter *d*_sp_ (10–90 nm), nanoparticle volume fraction *ϕ* (0.01–0.09), Rayleigh number *Ra* (10^3^–10^5^), Darcy number *Da* (10^−4^–10^−2^), porosity *ε* (0.1–0.9), and radius ratio *RR* (1.1–10) on the isotherms and streamlines, the local Nusselt number (*Nu*_loc_), and the average Nusselt number (*Nu*_avg_).

### 4.1. Effects of Brownian Motion

**Figure 3:**
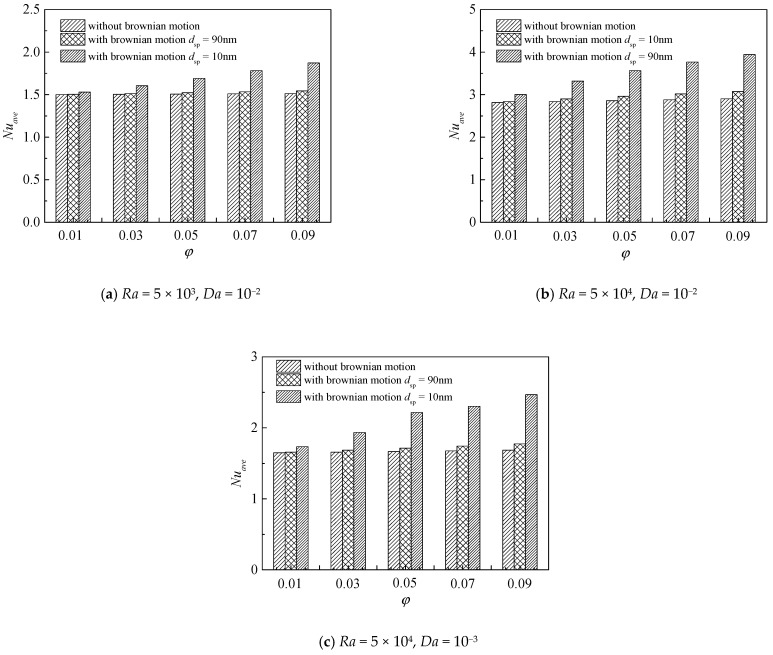
Effects of Brownian motion on the average Nusselt number (*Nu*_avg_) along the inner wall for different nanoparticle volume fractions (*ϕ*) and nanoparticle diameters (*d*_sp_) at *ε* = 0.5, *RR* = 2, and (**a**) *Ra* = 5 × 10^3^, *Da* = 10^−2^, (**b**) *Ra* = 5 × 10^4^, *Da* = 10^−2^, (**c**) *Ra* = 5 × 10^4^, *Da* = 10^−3^.

In this section, we have investigated the effect of Brownian motion on the heat transfer characteristics using two applied models in Table 2. One ignores the Brownian motion of the nanoparticles. The other takes Brownian motion into consideration and the modified effective thermal conductivity and effective dynamic viscosity are adopted. Figure 3 presents the effect of Brownian motion on the average Nusselt number along the inner wall under different parameters. From Figure 3a, the average Nusselt number increases generally as Brownian motion is considered. With the increase of nanoparticle volume fraction, the influence of Brownian motion becomes more noticeable. In addition, with the decrease of nanoparticle diameter, the influence of Brownian motion becomes more remarkable. For instance, when *Ra* = 5 × 10^3^, *Da* = 10^−2^, *d*_sp_ = 90 nm, *Nu*_avg_ with Brownian motion increased by 0.21% compared with that without Brownian motion at *ϕ* = 0.01, while the growth rate is 2.7% at *ϕ* = 0.09. When *Ra* = 5 × 10^3^, *Da* = 10^−2^, *d*_sp_ = 10 nm, *Nu*_avg_ with Brownian motion increased by 1.98% compared with that without Brownian motion at *ϕ* = 0.01, while the growth rate is 23.76% at *ϕ* = 0.09. Comparing Figure 3a,b and Figure 3b with Figure 3c, the effect of Brownian motion becomes more remarkable with the increase of Rayleigh number and the decrease of Darcy number. For example, when *Da* = 10^−2^, *ϕ* = 0.09, *d*_sp_ = 10 nm, *Nu*_avg_ with Brownian motion increased by 23.76% compared with that without Brownian motion at *Ra* = 5 × 10^3^, while the growth rate is 35.92% at *Ra* = 5 × 10^4^. When *Ra* = 5 × 10^4^, *ϕ* = 0.09, *d*_sp_ = 10 nm, *Nu*_avg_ with Brownian motion increased by 35.92% compared with that without Brownian motion at *Da* = 10^−2^, while the growth rate is 46.29% at *Da* = 10^−3^. Therefore, the effect of Brownian motion on the natural convection heat transfer of the nanofluid should be considered. Furthermore, the positive effect of Brownian motion on the overall heat transfer rate is different at different parameters.

### 4.2. Effects of Nanoparticle Volume Fraction

**Figure 4:**
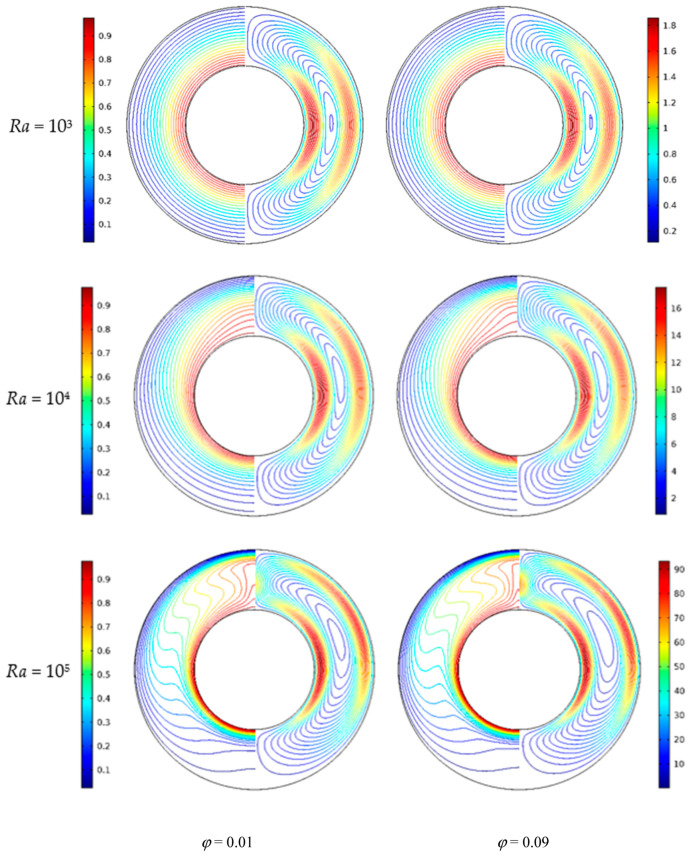
Isotherms (left) and streamlines (right) for Cu–water nanofluid for different nanoparticle volume fractions (*ϕ*) and Rayleigh numbers (*Ra*) at *d*_sp_ = 50 nm, *Da* = 0.01, *ε* = 0.5, and *RR* = 2.

**Figure 5:**
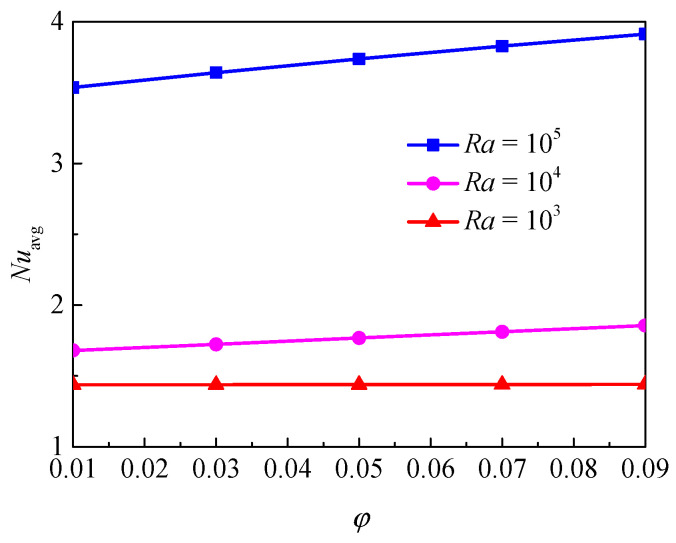
Average Nusselt number (*Nu*_avg_) of the inner wall for different nanoparticle volume fractions (*ϕ*) and Rayleigh numbers (*Ra*) at *Da* = 10^−2^, *d*_sp_ = 50 nm, *ε* = 0.5, and *RR* = 2.

Figure 4 shows the isotherms and streamlines for different nanoparticle volume fraction and Rayleigh number at *Da* = 10^−2^, *d*_sp_ = 50 nm, *ε* = 0.5, and *RR* = 2. The color scales on the left represent the dimensionless temperature and those on the right represent the dimensionless velocity, as in other sections of the article. From Figure 4, for *Ra* = 10^3^, the isotherms have a uniform distribution; this is because the buoyancy force is weak compared with the viscous force, and it indicates that the heat transfer in the annulus is dominated by thermal conduction. The effect of volume fraction on the isotherms is weak. For *Ra* = 10^4^, a slight thermal disturbance appeared, which indicates that the flow is enhanced and the transition from conduction to natural convection takes place. In this case, the effect of volume fraction becomes more important. For *Ra* = 10^5^, the isotherms are almost horizontally distributed, especially when *ϕ* = 0.9, which means that the natural convection heat transfer turns out to be more significant and the effect of volume fraction is more pronounced. With the increase of Rayleigh number, the streamlines become denser near the walls and the cell becomes bigger and has a tendency to move upward due to the enhanced buoyance force. In addition, for *Ra* = 10^3^ and *Ra* = 10^4^, the effect of volume fraction on the streamlines is very weak, while for *Ra* = 10^5^, the effect is more pronounced. Figure 5 displays the effect of volume fraction on the overall heat transfer rate along the inner wall at different Rayleigh numbers. The figure shows that an increase in volume fraction leads to heat transfer enhancement for all considered Rayleigh numbers, and the effect of volume fraction is more pronounced when the Rayleigh number is high.

**Figure 6:**
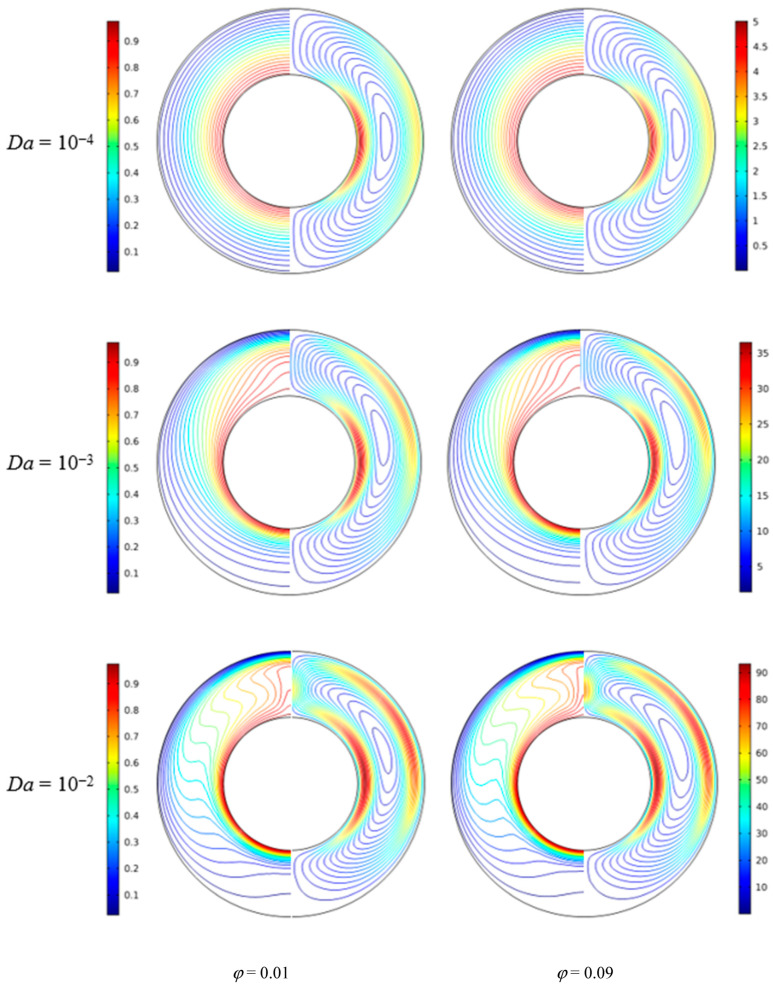
Isotherms (left) and streamlines (right) for Cu–water nanofluid for different nanoparticle volume fractions (*ϕ*) and Darcy numbers (*Da*) at *Ra*= 10^5^, *d*_sp_ = 50 nm, *ε* = 0.5, and *RR* = 2.

**Figure 7:**
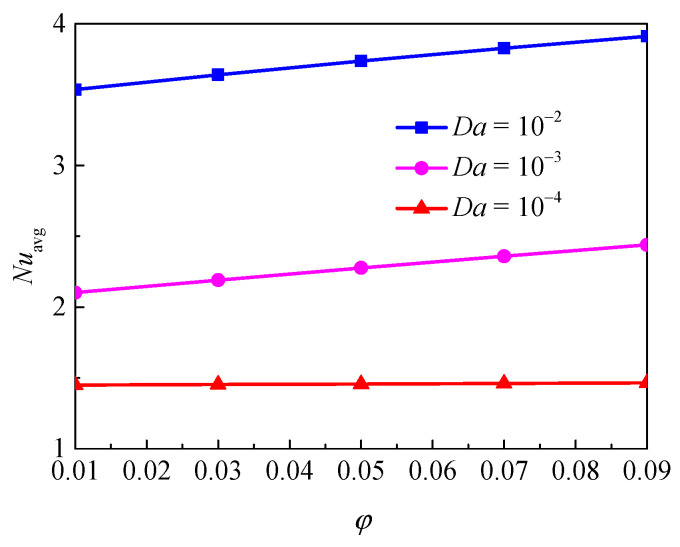
Average Nusselt number (*Nu*_avg_) of the inner wall for different nanoparticle volume fractions (*ϕ*) and Darcy numbers (*Da*) at *Ra*= 10^5^, *d*_sp_ = 50 nm, *ε* = 0.5, and *RR* = 2.

Figure 6 illustrates the isotherms and streamlines for different nanoparticle volume fraction and Darcy number at *d*_sp_ = 50 nm, *Ra* = 10^5^, *ε* = 0.5, and *RR* = 2. From Figure 6, for *Da* = 10^−4^, the isotherms have a uniform distribution due to the low permeability, and it indicates that the heat transfer in the annulus is dominated by thermal conduction. The effect of volume fraction on the isotherms is slight. For *Da* = 10^−3^, due to the enhanced permeability, the flow in the annulus is strengthened, and the transition from conduction to natural convection takes place. In this case, the effect of volume fraction becomes more important. For *Da* = 10^−2^, the isotherms are almost horizontally distributed, especially when *ϕ* = 0.9, which means the natural convection heat transfer plays a significant role and the effect of volume fraction is more pronounced. With the increase of Darcy number, the streamlines become denser near the walls and the cells become bigger and have a tendency to move upward due to the enhanced flow. In addition, for *Da* = 10^−4^ and *Da* = 10^−3^, the effect of volume fraction on the streamlines is very weak, while for *Da* = 10^−2^, the effect is more pronounced. Figure 7 displays the effect of volume fraction on the overall heat transfer rate along the inner wall at different Darcy numbers. The figure shows that an increase in volume fraction leads to heat transfer enhancement for all considered Darcy numbers and the effect of volume fraction is increased with the increase of Darcy number.

**Figure 8:**
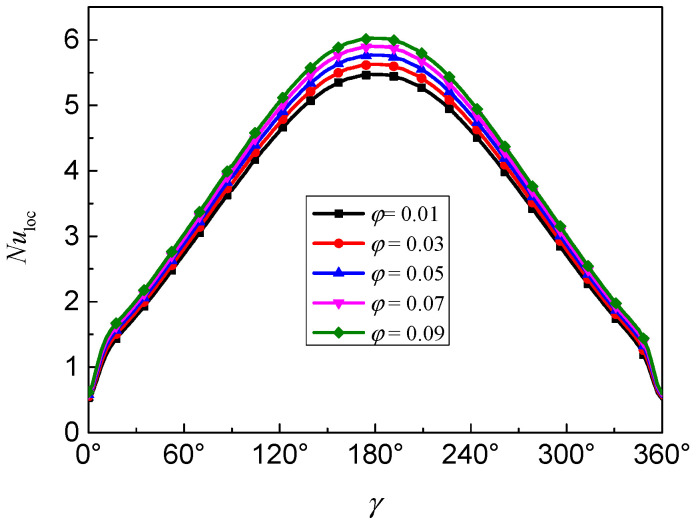
Local Nusselt number (*Nu*_loc_) along the inner wall for different nanoparticle volume fractions (*ϕ*) at *Ra* = 10^5^, *Da* = 10^−2^, *d*_sp_ = 50 nm, *ε* = 0.5, and *RR* = 2.

Figure 8 presents the evolution of the local Nusselt number along the inner wall for different nanoparticle volume fractions at *Ra* = 10^5^, *Da* = 10^−2^, *d*_sp_ = 50 nm, *ε* = 0.5, and *RR* = 2. The increase of *Nu*_loc_ in the whole region means that the local heat transfer rate is enhanced. It can be found that the maximum *Nu*_loc_ occurred at *γ* = 180°, which means the natural convection heat transfer is more intense in the bottom half of the inner wall. In addition, the heat transfer is enhanced with the increase of volume fraction.

### 4.3. Effects of Nanoparticle Diameter

**Figure 9:**
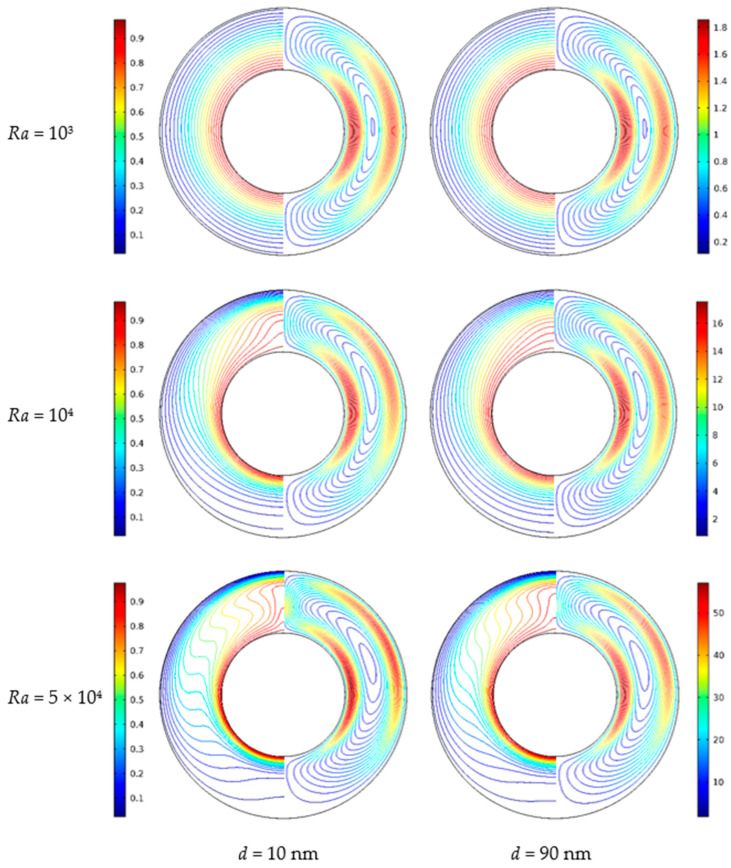
Isotherms (left) and streamlines (right) for Cu–water nanofluid for different nanoparticle diameters (*d*_sp_) and Rayleigh numbers (*Ra*) at *Da*= 10^−2^, *ϕ* = 0.05, *ε* = 0.5, and *RR* = 2.

**Figure 10:**
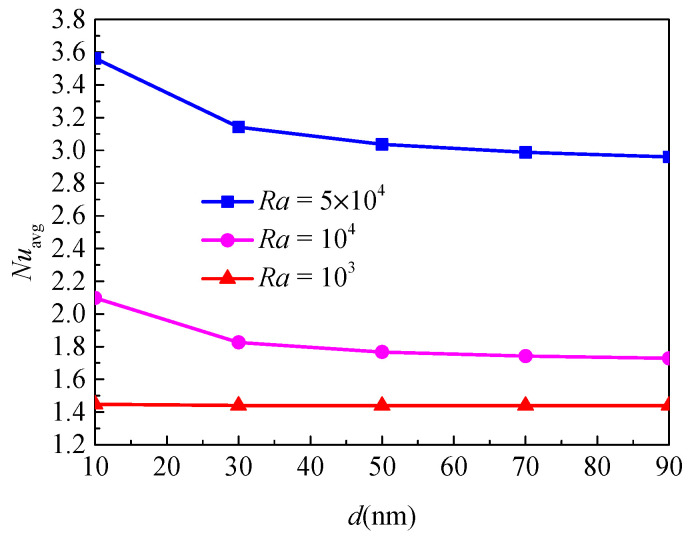
Average Nusselt number (*Nu*_avg_) of the inner wall for different nanoparticle diameters (*d*_sp_) and Rayleigh numbers (*Ra*) at *Da* = 10^−2^, *ϕ* = 0.05, *ε* = 0.5, and *RR* = 2.

Figure 9 shows the isotherms and streamlines for different nanoparticle diameters and Rayleigh numbers at *Da* = 10^−2^, *ϕ* = 0.05, *ε* = 0.5 and *RR* = 2. From Figure 9, for *Ra* = 10^3^, the isotherms have a uniform distribution due to the weak buoyancy force, and it indicates that the heat transfer in the annulus is dominated by thermal conduction. The nanoparticle diameter has a weak effect on the isotherms. For *Ra* = 10^4^, the natural convection heat transfer is strengthened due to the enhanced buoyancy force. The isotherms near the top half of the inner wall are disturbed, and the change of the isotherms distribution is more remarkable at *d*_sp_ = 10 compared with *d*_sp_ = 90. For *Ra* = 5 × 10^4^, the isotherms are almost horizontally distributed, especially when *d*_sp_ = 10, which means that the natural convection dominates the heat transfer and the effect of the nanoparticle diameter is more pronounced. In addition, for *Ra* = 10^3^ and *Ra* = 10^4^, the effect of nanoparticle diameter on the streamlines is very weak, while for *Ra* = 10^5^, the effect is more pronounced, and the cell becomes bigger and has a tendency to move upward. Figure 10 displays the effect of nanoparticle diameter on the overall heat transfer rate along the inner wall at different Rayleigh numbers. The figure shows that an increase in the nanoparticle diameter leads to reduced heat transfer for all considered Rayleigh numbers. For *Ra* = 10^3^, the effect of nanoparticle diameter is less pronounced. For *Ra* = 5 × 10^4^, the effect of nanoparticle diameter is remarkable, especially when it is at a low value. For instance, *Nu*_avg_ decreased by 11.79% from *d*_sp_ = 10 to *d*_sp_ = 30 and decreased by 5.81% from *d*_sp_ = 30 to *d*_sp_ = 90 at *Ra* = 5 × 10^4^, while *Nu*_avg_ decreased by 0.48% from *d*_sp_ = 10 to *d*_sp_ = 30 and decreased by 0.12% from *d*_sp_ = 30 to *d*_sp_ = 90 at *Ra* = 10^4^.

**Figure 11:**
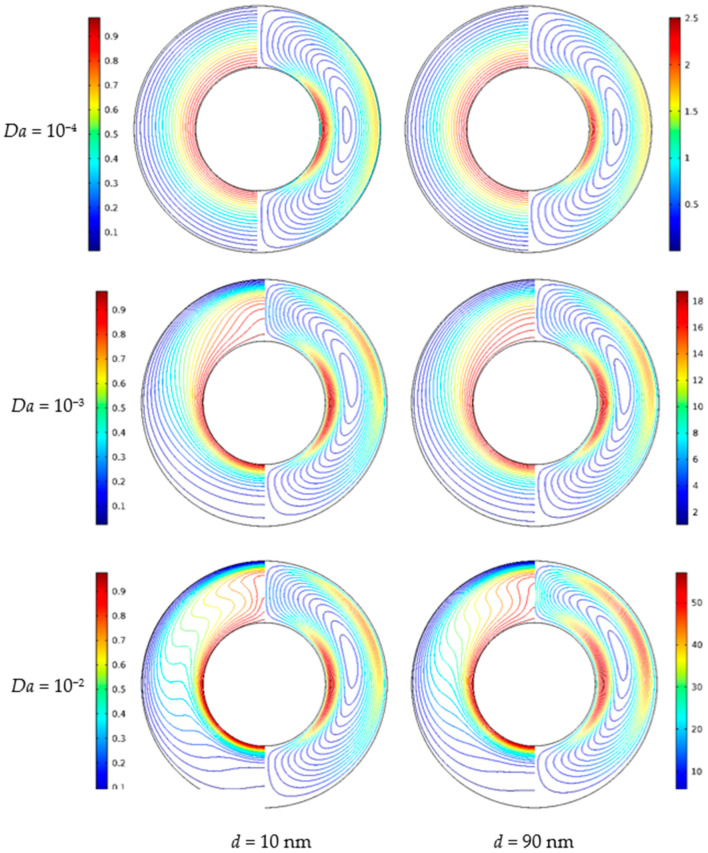
Isotherms (left) and streamlines (right) for Cu–water nanofluid for different nanoparticle diameters (*d*_sp_) and Darcy numbers (*Da*) at *Ra* = 5 × 10^4^, *ϕ* = 0.05, *ε* = 0.5, and *RR* = 2.

**Figure 12:**
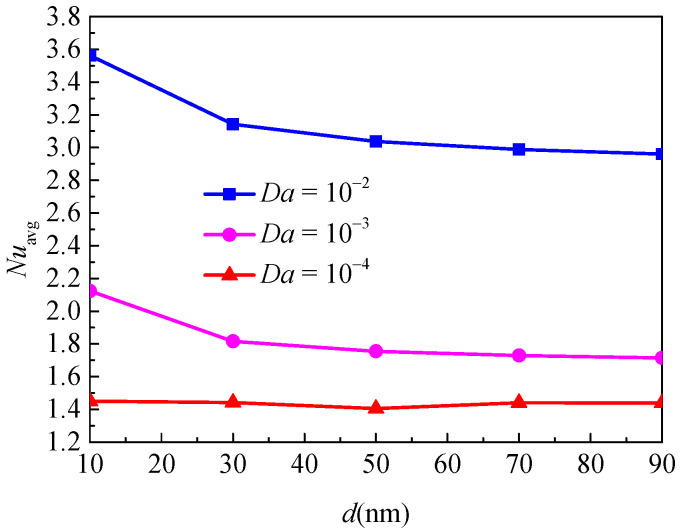
Average Nusselt number (*Nu*_loc_) of the inner wall for different nanoparticle diameters (*d*_sp_) and Darcy numbers (*Da*) at *Ra* = 5 × 10^4^, *ϕ* = 0.05, *ε* = 0.5, and *RR* = 2.

Figure 11 illustrates the isotherms and streamlines for different nanoparticle diameter and Darcy numbers at *Ra* = 5 × 10^4^, *ϕ* = 0.05, *ε* = 0.5, and *RR* = 2. From Figure 11, for *Da* = 10^−4^, the isotherms have a uniform distribution due to the low permeability, which indicates that the flow is weak and thermal conduction plays a leading role. The effect of nanoparticle diameter on the heat transfer is weak. For *Da* = 10^−3^, due to the enhanced permeability, the natural convection heat transfer is strengthened. For *Da* = 10^−2^, the isotherms are almost horizontally distributed, especially when *d*_sp_ = 10, which means that the natural convection heat transfer plays a significant role and the effect of nanoparticle diameter is more pronounced. In addition, for *Da* = 10^−4^ and *Da* = 10^−3^, the effect of nanoparticle diameter on the streamlines is very weak, while for *Da* = 10^−2^, the effect is more pronounced; the cell becomes bigger and has a tendency to move upward. Figure 12 displays the effect of the nanoparticle diameter on the overall heat transfer rate along the inner wall at different Darcy numbers. The figure shows that an increase in the nanoparticle diameter leads to heat transfer enhancement for all considered Darcy numbers, and it can be found that the effect of nanoparticle diameter is increased with the increase of the Darcy number.

**Figure 13:**
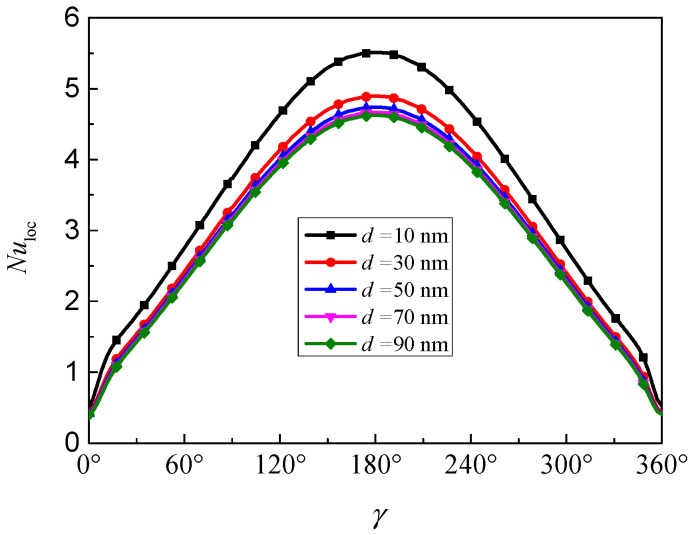
Local Nusselt number (*Nu*_loc_) along the inner wall for different nanoparticle diameters (*d*_sp_) at *Ra* = 10^5^, *Da* = 10^−2^, *ϕ* = 0.05, *ε* = 0.5, and *RR* = 2.

Figure 13 presents the evolution of the local Nusselt number along the inner wall for different nanoparticle diameters at *Ra* = 10^5^, *Da* = 10^−2^, *ϕ* = 0.05, *ε* = 0.5, and *RR* = 2. It can be found that the local Nusselt number has a symmetrical distribution due to the symmetrical geometry and boundary conditions, and the maximum *Nu*_loc_ occurred at *γ*= 180°, which means that the natural convection heat transfer is more intense in the bottom half of the inner wall. Furthermore, the local Nusselt number is increased with the decrease of nanoparticle diameter, which indicates that the nanoparticle diameter at high value will weaken the natural convection heat transfer.

### 4.4. Effects of Porosity

**Figure 14:**
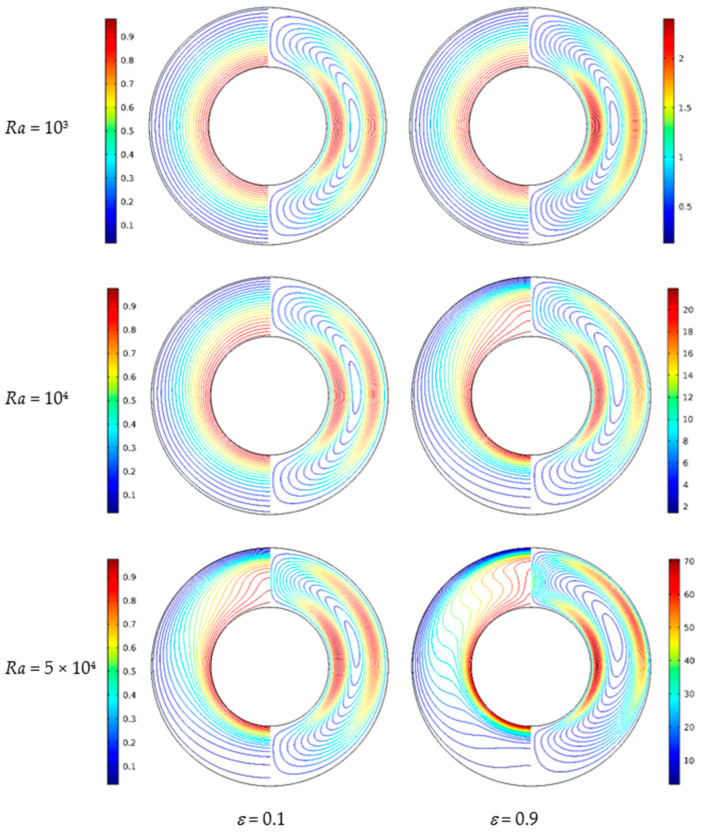
Isotherms (left) and streamlines (right) for Cu–water nanofluid for different porosity (*ε*) and Rayleigh numbers (*Ra*) at *Da* = 10^−2^, *ϕ* = 0.05, *d*_sp_ = 50 nm, and *RR* = 2.

**Figure 15:**
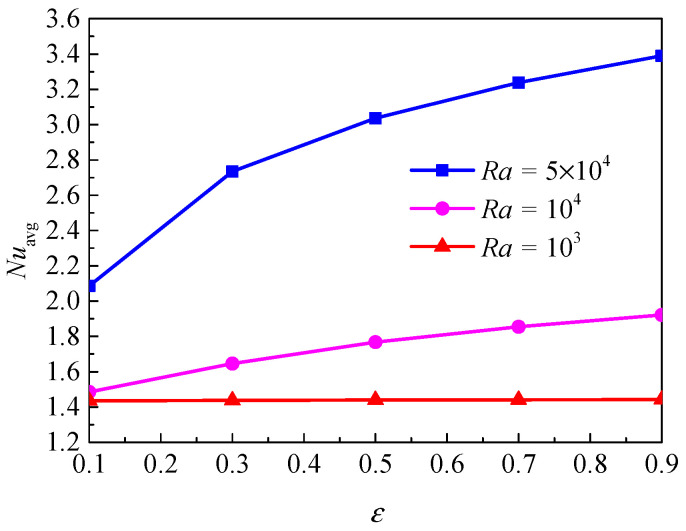
Average Nusselt number (*Nu*_avg_) of the inner wall for different porosity (*ε*) and Rayleigh numbers (*Ra*) at *Da* = 10^−2^, *ϕ* = 0.05, *d*_sp_ = 50 nm, and *RR* = 2.

Figure 14 shows the isotherms and streamlines for different porosity and Rayleigh numbers at *d*_sp_ = 50 nm, *Da* = 10^−2^, and *RR* = 2. From Figure 14, for *Ra* = 10^3^, the isotherms have a uniform distribution, and the isotherms are almost unchanged when the porosity increased from *ε* = 0.1 to *ε* = 0.9. This is because at low Rayleigh numbers, the buoyancy force is very weak, and the heat transfer in the annulus is dominated by thermal conduction. The porosity has a slight effect on the isotherms. For *Ra* = 10^4^, the isotherms have a similar trend with that for *Ra* = 10^3^ at *ε* = 0.1, while the isotherms have an obvious change at *ε* = 0.9. For *Ra* = 5 × 10^4^, the natural convection heat transfer plays a leading role due to the enhanced flow, and the heat transfer is more intense at *ε* = 0.9 compared with *ε* = 0.1. The streamlines have a similar distribution, but some details are different. For *Ra* = 10^3^, the streamlines have little change at *ε* = 0.1 and *ε* = 0.9. For *Ra* = 10^4^ and *Ra* = 5 × 10^4^, it can be found the cells become bigger and have a tendency to move upward when the porosity increases. Figure 15 displays the effect of porosity on the overall heat transfer rate along the inner wall at different Rayleigh numbers. It can be found that a continuous increase of the overall heat transfer rate occurs with the increase of porosity for all considered Rayleigh numbers and the effect of porosity is more pronounced at high Rayleigh numbers.

**Figure 16:**
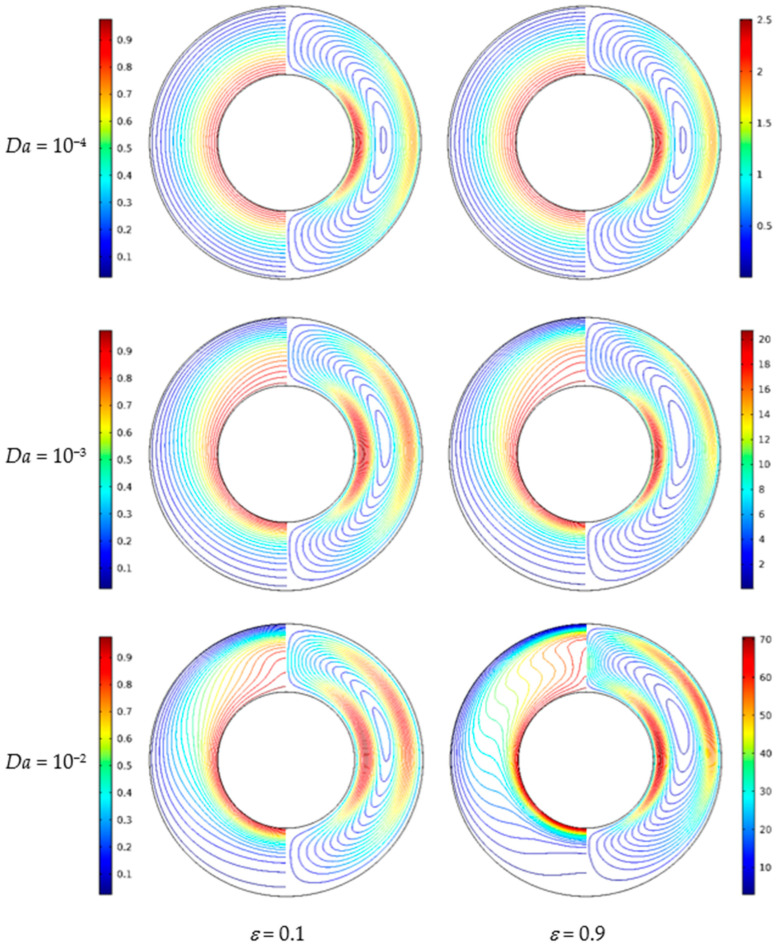
Isotherms (left) and streamlines (right) for Cu–water nanofluid for different porosity (*ε*) and Darcy numbers (*Da*) at *Ra* = 5 × 10^4^, *ϕ* = 0.05, *d*_sp_ = 50 nm, and *RR* = 2.

**Figure 17:**
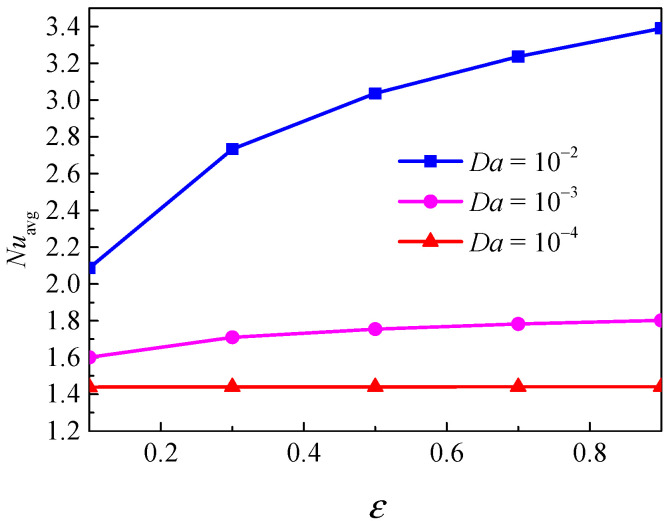
Average Nusselt number (*Nu*_avg_) of the inner wall for different porosity (*ε*) and Darcy numbers (*Da*) at *Ra* = 5 × 10^4^, *ϕ* = 0.05, *d*_sp_ = 50 nm, and *RR* = 2.

Figure 16 displays the isotherms and streamlines for different porosity and Darcy numbers at *Ra* = 5 × 10^4^, *ϕ* = 0.05, *d*_sp_ = 50 nm, and *RR* = 2. From Figure 16, for *Da* = 10^−4^, the isotherms have a uniform distribution at *ε* = 0.1 and *ε* = 0.9; the reason is that the flow is constrained by the low permeability. In this case, porosity has little effect on the isotherms. For *Da* = 10^−3^, due to the enhanced permeability, the flow in the annulus is strengthened, and a disturbance occurs in the isotherms. For *Da* = 10^−2^, the isotherms at *ε* = 0.1 and *ε* = 0.9 have a remarkable difference. The isotherms are almost horizontally distributed at *ε* = 0.9, which means that the overall heat transfer is dominated by natural convection. For *Da* = 10^−4^, the streamlines are almost the same at *ε* = 0.1 and *ε* = 0.9. For *Da* = 10^−3^ and *Da* = 10^−2^, the cell becomes bigger and has a tendency to move upward when porosity increases from *ε* = 0.1 to *ε* = 0.9. Figure 17 displays the effect of porosity on the overall heat transfer rate along the inner wall at different Darcy number. The figure shows that an increase in porosity leads to heat transfer enhancement for all considered Darcy numbers, and the effect of porosity is more pronounced at high Darcy numbers.

**Figure 18:**
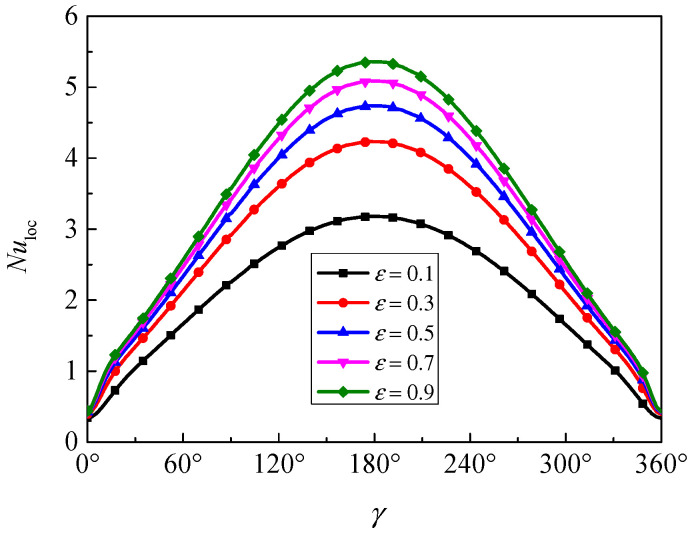
Local Nusselt number (*Nu*_loc_) along the inner wall for different porosity (*ε*) at *Ra* = 5 × 10^4^, *Da* = 10^−2^, *ϕ* = 0.05, *d*_sp_ = 50 nm, and *RR* = 2.

Figure 18 presents the evolution of the local Nusselt number along the inner wall for different porosity at *Ra* = 5 × 10^4^, *Da* = 10^−2^, *ϕ* = 0.05, *ε* = 0.5, *d*_sp_ = 50 nm, and *RR* = 2. The local Nusselt number has a symmetrical distribution due to the symmetrical geometry and boundary conditions, and the maximum *Nu*_loc_ occurred at *γ* = 180°. Furthermore, the local Nusselt number is increased with the increase of porosity, so it can be concluded that the porosity has a positive effect on the overall heat transfer rate.

### 4.5. Effects of Radius Ratio

**Figure 19:**
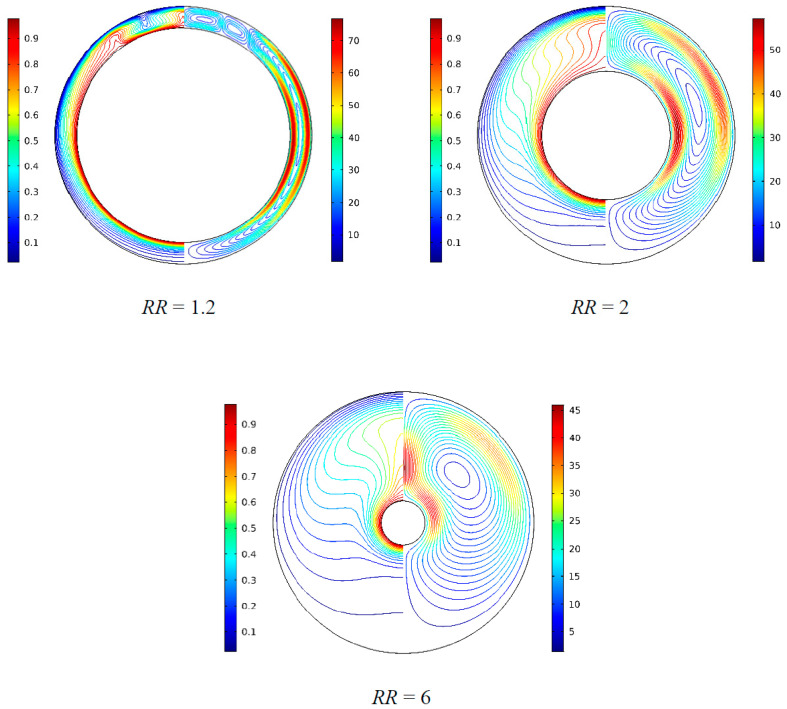
Isotherms (left) and streamlines (right) for Cu–water nanofluid for different radius ratios (*RR*) at *Ra* = 5 × 10^4^, *Da* = 10^−2^, *ϕ* = 0.05, *d*_sp_ = 50 nm, and *ε* = 0.5.

**Figure 20:**
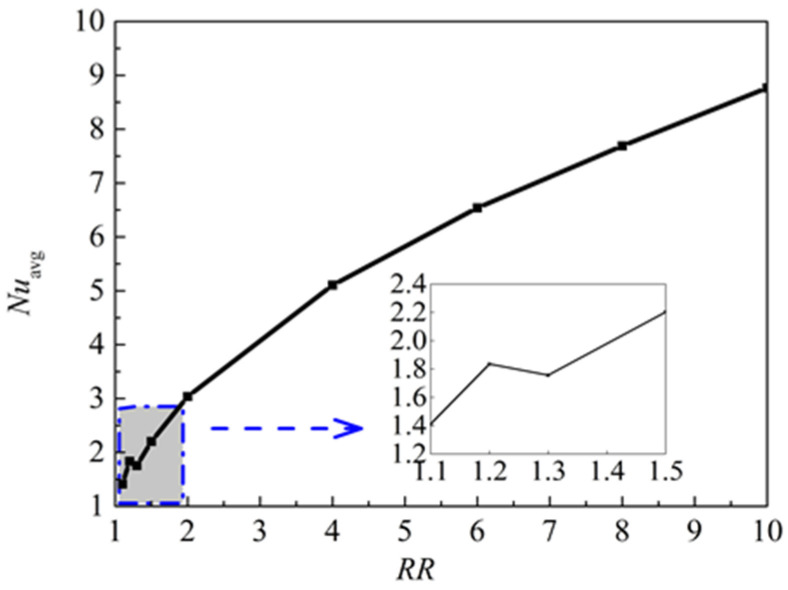
Average Nusselt number (*Nu*_avg_) of the inner wall for different radius ratios (*RR*) at *Ra* = 5 × 10^4^, *Da* = 10^−2^, *ϕ* = 0.05, *d*_sp_ = 50 nm, and *ε* = 0.5.

Figure 19 illustrates the isotherms and streamlines for different radius ratio at *Ra* = 5 × 10^4^, *Da* = 10^−2^, *ϕ* = 0.05, *d*_sp_ = 50 nm, and *ε* = 0.5. The radius ratio is an important parameter that affects the fluid flows and natural convection heat transfer according the previous research [10,11,12]. From Figure 19, when the radius ratio increased from *RR* = 1.2 to *RR*= 6, the flow is strengthened, a main cell is formed in the middle of the annulus, and the cell becomes bigger and has a tendency to move upward. It is worth noting that the color scales on the right have a downward trend with the increase of *RR*, which is inverse to the above conclusion. In fact, the reason is that the change of *RR* leads to a change of the characteristic length (*r*_o_–*r*_i_). The isotherms distribution is changed from vertical to horizontal, which means that the heat transfer is dominated by natural convection. When *RR* = 1.2, multicellular flow structures are formed. Figure 20 displays the effect of radius ratio on the overall heat transfer rate along the inner wall. It can be found that heat transfer in the annulus is enhanced with the increase of radius ratio from *RR* = 1.3 to *RR* = 6, but a fall occurred in the range of *RR* = 1.2 to *RR* = 1.3. A conclusion can be drawn that a bifurcation point exists when the radius ratios is in the range of *RR* = 1.2 to *RR* = 1.3 for the considered parameters.

## 5. Conclusions

Natural convection heat transfer in a porous annulus filled with a Cu-Nanofluid has been investigated numerically. The effects of Brownian motion, solid volume fraction, nanoparticle diameter, Rayleigh number, Darcy number, porosity on the flow pattern, temperature distribution, and heat transfer characteristics are discussed in detail. The following conclusions could be drawn:

(1) Brownian motion should be considered in the natural convection heat transfer of a nanofluid.

The effect of Brownian motion becomes more remarkable with the increase of Rayleigh number and nanoparticle volume fraction, while it is less pronounced with the increase of Darcy number and nanoparticle diameter.

(2) The increase of nanoparticle volume fraction results in an improvement of the overall heat transfer rate, and the effect is more remarkable when the Rayleigh and Darcy numbers are at high value.

(3) Increasing the nanoparticle diameter has a negative effect on the overall heat transfer rate and the effect has a limit when the nanoparticle diameter reaches a high value.

(4) The porosity affects the flow pattern, temperature distribution, and heat transfer rate. The flow motion is limited when the porosity is too low. The heat transfer rate is strengthened with the increase of porosity, especially with high Rayleigh and Darcy numbers.

(5) The radius ratio has a significant influence on the isotherms, streamlines, and heat transfer rate. The rate is greatly enhanced with the increase of radius ratio. Additionally, when the radius ratio is too low, multicellular flow structures are formed, and a bifurcation point exists.

## Figures and Tables

**Table 1:** Thermal physical properties of the base fluid (water), nanoparticle (Cu), and solid of the porous medium (glass balls).

Physical Properties	Base Fluid (Water)	Nanoparticle (Cu)	Porous (Glass Balls)
*ρ* [kg/m^3^]	997.1	8933	2700
*c*_p_ [J/(kg·K)]	4179	385	840
*k* [W/(m·K)]	0.613	76.5	1.05
*μ* [kg/(m·s)]	0.001003	-	-
*β* × 10^5^ [1/K]	21	1.67	0.9

**Table 2:** Applied models for thermophysical properties of the nanofluid with or without Brownian motion.

Physical Properties	Applied Model	
	Without Brownian Motion	With Brownian Motion
*k* [W/(m·K)]	$\frac{k_{\mathrm{nf}}}{k_{\mathrm{bf}}}=\frac{k_{\mathrm{sp}}+2k_{\mathrm{bf}}-2\varphi (k_{\mathrm{bf}}-k_{\mathrm{sp}})}{k_{\mathrm{sp}}+2k_{\mathrm{bf}}+\varphi (k_{\mathrm{bf}}-k_{\mathrm{sp}})}$	$k_{\mathrm{nf}}=k_{\mathrm{bf}}\frac{k_{\mathrm{sp}}+2k_{\mathrm{bf}}-2\varphi (k_{\mathrm{bf}}-k_{\mathrm{sp}})}{k_{\mathrm{sp}}+2k_{\mathrm{bf}}+\varphi (k_{\mathrm{bf}}-k_{\mathrm{sp}})}+C{\left(\rho c_{p}\right)}_{\mathrm{nf}}\frac{2k_{b}T_{o}}{\pi \mu_{\mathrm{nf}}d_{\mathrm{sp}}{}^{2}}\varphi d_{\mathrm{sp}}$
*μ* [kg/(m·s)]	*μ*_nf_ = *μ*_bf_/(1 − *ϕ*)^2.5^	*μ*_nf_ = *μ*_bf_/[1 − 34.87(*d*_s__p_/*d*_bf_)^−0.3^*ϕ*^1.03^];
*ρ* [kg/m^3^]	(*ρc*_p_)_nf_ = (1 − *ϕ*)(*ρc*_p_)_bf_ + *ϕ*((*ρ* *c*_p_)_sp_	
*c*_p_ [J/(kg·K)]	*ρ*_nf_(*T*) = (1 − *ϕ*)*ρ*_bf_(*T*) + *ϕρ*_s__p_;	

*C* = 3.6 × 10^4^; *k*_b_ = 1.38065 × 10^−23^ J K^−1^, *d*_bf_ = 0.1[6*M*/(*Nπρ*_m_)]^1/3^: *M* = 0.018 kg mol^−1^, *N* = 6.022 × 10^23^ mol^−1^, *ρ*, (*ρc*_p_) and *μ* denote the density, heat capacitance, and dynamic viscosity, respectively, *k* is the thermal conductivity, *ϕ* is the nanoparticle volume fraction, and the subscripts bf, sp, and nf designate the base fluid, nanoparticle, and nanofluid.

**Table 3:** Comparison of the average Nusselt number for Cu nanofluid at different levels when *ϕ* = 0.5, *d*_sp_ = 50 nm, *Ra* = 10^5^, *ε* = 0.5, and *Da* = 10^−2^.

Level	Number of Elements	Minimum Quality	Average Quality	*Nu*_avg_
normal	894	0.4632	0.7779	2.2466
fine	1344	0.4938	0.8284	2.2632
finer	1882	0.5084	0.8305	2.2668
extra fine	6394	0.5276	0.8424	2.2766
extremely fine	17858	0.5053	0.8466	2.2769

**Table 4:** Comparison of the average Nusselt number with those of Singh et al. [37] for different Rayleigh number when *φ* = 0, *ε* = 0.4, 0.9, *Da* = 10^−2^, and *Pr* = 1.

Darcy Number (*Da*)	Reyleigh Number (*Ra*)	Average Nusselt Number (*Nu*_avg_)					
		*ε* = 0.4			*ε* = 0.9		
		Ref [37]	Present Work	Diff(%)	Ref [37]	Present Work	Diff(%)
10^−2^	10^5^	2.983	3.008	0.84	3.91	3.95	1.3
	10^4^	1.408	1.408	0	1.64	1.66	1.2
	10^3^	1.01	1.01	0	1.023	1.028	0.5

## Data Availability

Data available in a publicly accessible repository.

## Nomenclature

*RR*	radius ratio
*c*_p_	thermal capacity, J/(kg × K)
*d*	nanoparticle diameter, m
*Da*	Darcy number
*g*	acceleration due to gravity, m/s^2^
*k*	thermal conductivity, W/(m × K)
*K*	medium permeability, m^2^
*Nu*	Nusselt number
*p*	pressure, Pa
*P*	dimensionless pressure
*Pr*	Prandtl number
*r*	radius, m
*Ra*	Rayleigh number
*T*	temperature, K
(*u*, *v*)	velocity, m/s
(*U*, *V*)	dimensionless velocity
(*x*, *y*)	cartesian coodinates
(*X*, *Y*)	dimensionless cartesian coodinates
Greek symbols	
*α*	thermal diffusivity, m^2^/s
*β*	thermal expansion coefficient, K^−1^
*γ*	angle, deg
*ε*	porosity
*θ*	dimensionless temperature
*ρ*	density, Kg/m^3^
*μ*	dynamic viscosity, kg/(m × s)
*ν*	kinematic viscosity, m^2^/s
*ϕ*	nanoparticle volume fraction
Subscripts	
avg	average
bf	base fluid
i, o	inner, outer
loc	local
mnf	porous medium filled with nanofluid
nf	nanofluid
s	porous medium
sp	nanoparticle
